# Introducing an Effective Dementia Training for Community Health Workers

**DOI:** 10.1093/geroni/igaf122.3182

**Published:** 2025-12-31

**Authors:** Kerstin Reinschmidt, Zahra Alhay, Keith Kleszynski, Lee Jennings

**Affiliations:** The University of Oklahoma Health Sciences Center, Oklahoma City, Oklahoma, United States; The University of Oklahoma Health Sciences Center, Oklahoma City, Oklahoma, United States; University of Oklahoma Health Campus, Oklahoma City, Oklahoma, United States; University of Oklahoma Health Sciences, Oklahoma City, Oklahoma, United States

## Abstract

Community health workers (CHWs) have been identified as a public health workforce that can help remedy the national dementia crisis by supporting individuals and communities to prevent and recognize dementia. CHWs who completed workforce-appropriate trainings to address dementia are likely to have a positive impact on healthy aging and living with dementia. The Oklahoma Dementia Care Network developed the Dementia Training for Community Health Workers by combining knowledge about CHWs in Oklahoma, national recommendations on the CHW scope of work, and evidence-based dementia care strategies. Utilizing the Kirkpatrick Model, we evaluated the training effectiveness with a focus on knowledge and skill acquisition, CHW training feedback, and application of knowledge and resources shared during the training. We conducted 15 trainings with 307 trainees between June 2020 and March 2024. Data was collected on pre/post knowledge, self-efficacy, and post-training feedback. Analyzing a subsample of trainees (50%; n = 154), we found that both knowledge and some of the self-efficacy items showed statistically significant improvements. Trainees were satisfied with the training content, format, and delivery. Responding to a follow-up survey, they shared their appreciation of the practical value of the training to their jobs. Our training has practical implications for workforce development as it prepares CHWs to address dementia within their broad scope of work. The training can be adapted by different communities using culturally relevant narratives that reflect unique values and experiences.

